# The lattice of trumping majorization for 4D probability vectors and 2D catalysts

**DOI:** 10.1038/s41598-018-21947-0

**Published:** 2018-02-27

**Authors:** Gustavo M. Bosyk, Hector Freytes, Guido Bellomo, Giuseppe Sergioli

**Affiliations:** 1 Instituto de Física La Plata, UNLP, CONICET, Facultad de Ciencias Exactas, 1900 La Plata, Argentina; 2 Università degli Studi di Cagliari, Via Is Mirrionis 1, I-09123 Cagliari, Italy; 3 Departamento de Matemática, UNR, CONICET, 2000 Rosario, Argentina; 4 CONICET-Universidad de Buenos Aires, Instituto de Investigación en Ciencias de la Computación (ICC), Buenos Aires, Argentina

## Abstract

The transformation of an initial bipartite pure state into a target one by means of local operations and classical communication and entangled-assisted by a catalyst defines a partial order between probability vectors. This partial order, so-called trumping majorization, is based on tensor products and the majorization relation. Here, we aim to study order properties of trumping majorization. We show that the trumping majorization partial order is indeed a lattice for four dimensional probability vectors and two dimensional catalysts. In addition, we show that the subadditivity and supermodularity of the Shannon entropy on the majorization lattice are inherited by the trumping majorization lattice. Finally, we provide a suitable definition of distance for four dimensional probability vectors.

## Introduction

Majorization is nowadays a well-established and powerful mathematical tool to compare probability vectors, which is applied in many and different fields as in economy, biology, physics among others (see *e.g*. ref.^1^ for an introduction to the topic). The spread applicability of majorization in the quantum realm emerges as a consequence of deep connections among majorization, partially ordered probability vectors, unitary matrices and the probabilistic structure of quantum mechanics (see *e.g*. refs^2–14^ for different applications of majorization in the quantum realm).

An interesting problem where majorization naturally arises is the interconversion of bipartite pure states by means of local operations and classical communication (LOCC). More precisely, this problem consists in two parties, Alice and Bob, that share an (initial) entangled pure-state, say $|x\rangle$ Their goal is to transform *x* into another entangled pure-state (target state), say $|y\rangle$, by applying LOCC. A celebrated result of Nielsen gives the necessary and sufficient condition that makes this entanglement transformation process possible^15^. Indeed, this process can be achieved if and only if there exists a majorization relation between the initial and target states, that is, $|x\rangle \mathop{\mathrm{\to }}\limits_{{\rm{LOCC}}}|y\rangle$ iff ${x}\,\prec \,{y}$, where $\prec$ denotes a majorization relation between the probability vectors formed by the Schmidt coefficients of the initial and target states, respectively. Notice that from the viewpoint of order theory, majorization gives a partial order among probability vectors with components sorted in nonincreasing order. Moreover, Cicalese and Vaccaro have shown that majorization has a more rich structure, indeed it is a lattice^16^. Recently, this lattice structure has been studied in connection with the problem of approximate entanglement transformations^17^ and the thermodynamic arrow of time in quantum and classical regimes^18^.

Since majorization is not a total order, there exist states $|x\rangle$ and $|y\rangle$ for which this transformation $|x\rangle \mathop{\mathrm{\to }}\limits_{{\rm{LOCC}}}|y\rangle$ is not possible. In order to overcome this situation, different protocols have been proposed, for instance: when the initial state has at least as many nonvanishing Schmidt coefficients as the target state, the transformation can be locally achieved with a non zero probability of success^19^; alternatively, a deterministic protocol has been proposed where the final state is the most approximate (in the sense of maximal fidelity between the final and target states) to the target one^20^. A third option, which is the one we are interested in, is the entangled-assisted LOCC (ELOCC) protocol introduced by Jonathan and Plenio in ref.^21^. The idea is to achieve the desired transformation with the help of an additional resource, say $|c\rangle$, which is an entangled state, so-called catalyst in analogy to a chemical process. A typical example is the following: let $|x\rangle =\sqrt{0.4}|00\rangle +\sqrt{0.4}|11\rangle +\sqrt{0.1}|22\rangle +\sqrt{0.1}|33\rangle$ and $|y\rangle =\sqrt{0.5}|00\rangle +\sqrt{0.25}|11\rangle +\sqrt{0.25}|22\rangle$ be initial and target states, respectively, for which can be shown that $|x\rangle \mathop{\mathrm{\nrightarrow }}\limits_{{\rm{LOCC}}}|y\rangle$. By appealing to the catalyst $|c\rangle =\sqrt{0.6}|44\rangle +\sqrt{0.4}|55\rangle$, the following transformation $|x\rangle \otimes |c\rangle \mathop{\mathrm{\to }}\limits_{{\rm{LOCC}}}|y\rangle \otimes |c\rangle$ can be achieved, without disturbing the catalyst. Let us notice that ELOCC defines a new partial order, which is called trumping majorization. Unlike what happens with the standard majorization relation, it is harder to obtain mathematical properties of trumping majorization. Most of the additional difficulties arise from the need to re-sort in nonincreasing order the components of the vector after the application of the catalyst. For that reason, there are not so many general analytical results about trumping majorization (see *e.g*. refs^22–26^ for some general results). On the other hand, some results have been provided by restricting the dimension of input vectors and/or the dimension of the catalyst (see *e.g*. refs^27–30^).

Here, we aim to study order properties of trumping majorization. Indeed, if trumping majorization gives or not a lattice structure is still an open question^31^. We will provide a positive answer to this question by restricting to the case of input probability vectors of dimension four and catalysts of dimension two. Clearly, this is not the most general case, but dimension four of probability vectors is the lowest dimension that one can consider in order to get something different to the standard majorization relation^21,22^. In addition, a catalyst of dimension two can represent a two-qubit state, which is the ubiquitous paradigm in several protocols of quantum information. Moreover, the study of the lattice properties of trumping majorization deserves attention as it could lead to a better understanding not only of the ELOCC transformations^21^, but also of catalytic coherence transformations^32,33^.

## Results

### Majorization and trumping majorization

In this Section, we review the definitions of majorization and trumping majorization and some related results.

Hereafter, let us consider the set of *d*-dimensional probability vectors whose components are sorted in nondecreasing order, namely

1 $${{\rm{\Delta }}}_{d}=\{({x}_{1},\ldots ,{x}_{d}):{x}_{i}\ge {x}_{i+1}\ge 0\,{\rm{and}}\,\sum _{i=1}^{d}{x}_{i}=1\}.$$

Majorization for probability vectors can be defined in the following way:

#### Definition 1.

*Let x and y be probability vectors in* Δ_*d*_. *Then, it is said that x is majorized by y, denoted as* $x\,\prec \,y$, *if and only if*,

2 $$\sum _{i=1}^{n}{x}_{i}\le \sum _{i=1}^{n}{y}_{i}\quad \,for\,all\,n\in \{1,\ldots ,d-1\}\mathrm{.}$$

In order to have a majorization relation between *x* and *y*, the *d* − 1 in equalities (2) have to be satisfied (notice that ${\sum }_{i=1}^{d}{x}_{i}={\sum }_{i=1}^{d}{y}_{i}$ is trivially satisfied, for that reason we discard this condition from the definition of majorization).

The majorization relation, $x\,\prec \,y$, can be posed in several equivalent ways (see *e.g*. ref.^1^). On one hand, $x\,\prec \,y$ iff there exists a double stochastic matrix *D*, i.e., *D*_*ij*_ ≥ 0 for all *i*, *j* and ${\sum }_{i=1}^{d}{D}_{ij}=1={\sum }_{j=1}^{d}{D}_{ij}$, such that *x* = *Dy*. On the other hand, $x\,\prec \,y$ iff ${\sum }_{i=1}^{d}\varphi ({x}_{i})\ge {\sum }_{i=1}^{d}\varphi ({y}_{i})$ for every concave function *ϕ*. This last inequality links the notion of majorization with the concepts of Schur-concavity and entropy.

#### Definition 2.

*Let* ${\rm{\Phi }}:{{\rm{\Delta }}}_{d}\mapsto {\mathbb{R}}$. *The function* Φ *is a Schur-concave function if it anti-preserves the majorization relation. That is*,

3 $$\,if\,x\,\prec \,y\Rightarrow {\rm{\Phi }}(x)\ge {\rm{\Phi }}(y),\,for\,all\,\,x,y\in {{\rm{\Delta }}}_{d}\mathrm{.}$$

The following very general family of entropies satisfies the Schur-concavity^14^,

4 $${H}_{(h,\varphi )}(x)=h(\sum _{i=1}^{d}\varphi ({x}_{i})),$$

where the entropic functionals $h:{\mathbb{R}}\mapsto {\mathbb{R}}$ and $\varphi :\,\mathrm{[0},\mathrm{1]}\mapsto {\mathbb{R}}$ are such that either: (i) *h* is increasing and *ϕ* is concave, or (ii) *h* is decreasing and *ϕ* is convex, together with the conventions *ϕ*(0) = 0 and *h*(*ϕ*(1)) = 0. Notice that Shannon, Rényi and Burg entropies, given by

5 $${H}_{1}(x)=-\sum _{i=1}^{d}{x}_{i}\,\mathrm{log}\,{x}_{i},$$

6 $${H}_{\alpha }(x)=\frac{{\rm{sign}}\alpha }{1-\alpha }\,\mathrm{log}\,\sum _{i=1}^{d}{x}_{i}^{\alpha }\,{\rm{for}}\,{\rm{all}}\,\alpha \in {\mathbb{R}}\backslash \mathrm{\{0},\mathrm{1\}}\,{\rm{and}}$$

7 $${H}_{{\rm{Burg}}}(x)=\frac{1}{d}\sum _{i=1}^{d}\,\mathrm{log}\,{x}_{i},$$

respectively, are Schur-concave and additive particular cases of Eq. (4).

From the viewpoint of order theory, majorization gives a partial order among probability vectors belonging to Δ_*d*_. This means that majorization is a binary relation that, for all *x*, *y*, *z* ∈ Δ_*d*_, satisfies:

(I) Reflexivity: $$x\,\prec \,x$$,
(II) Symmetry: if $x\,\prec \,y$ and $y\,\prec \,x$, then *x* = *y*,
(III) Transitivity: if $x\,\prec \,z$ and $z\,\prec \,y$, then $x\,\prec \,y$.

Remarkably enough, Cicalese and Vaccaro have shown that majorization is indeed a lattice^16^. This means that there exist the infimum (join) *x* ∧ *y* and the supremum (meet) *x* ∨ *y*. By definition, *x* ∧ *y* means that $x\,\wedge \,y\,\prec \,x$, $x\,\wedge \,y\,\prec \,y$ and $z\,\prec \,x\,\wedge \,y$ for all *z* such that $z\,\prec \,x$ and $z\,\prec \,y$. In a similar way, *x* ∨ *y* means that $x\,\prec \,x\,\vee \,y$, $y\,\prec \,x\,\vee \,y$ and $x\,\vee \,y\,\prec \,z$ for all *z* such that $x\,\prec \,z$ and $y\,\prec \,z$. The algorithms to obtain the infimum, *x* ∧ *y*, and the supremum, *x* ∨ *y*, are given in ref.^16^.

As we recall in the Introduction, majorization plays a crucial role in the problem of interconversion of bipartite pure states by means of LOCC^15^. On the other hand, trumping majorization plays an analogue role in ELOCC protocols^21^. The definition of trumping majorization is based on the standard one and tensor products between probability vectors.

#### Definition 3.

*Let x and y be probability vectors in* Δ_*d*_. *Then, it is said that x is trumped by y, denoted as* $x\,{\prec }_{T}\,y$, *if and only if there exist a catalyst c* ∈ Δ_*d*′_ (*with d*′ *an arbitrary dimension*) *such that* $x\,\otimes \,c\,\prec \,y\,\otimes \,c$.

Notice that there are (at least) two difficulties in order to realize whether, given two probability vectors, there is a trumping majorization relation between them. On one hand, the catalyst is a probability vector of arbitrary dimension. On the other hand, when a particular catalyst is chosen, one has to re-sort the components of the corresponding probability vectors obtained after the tensor products. How to manage with these two issues makes it very hard to obtain a general characterization of the probability vectors that are trumped. However, there are some results that will be useful for us. In first place, trumping majorization can be posed in an equivalent way in terms of a set of inequalities related to Shannon, Rényi and Burg entropies^23–26^. More precisely,

#### Theorem 1.

*Let x*, *y* ∈ Δ_*d*_*, such that x*  ≠ *y and some of them has full rank. Then, one has*

8 $$x\,{\prec }_{T}\,y\quad iff\quad {H}_{{\rm{Burg}}}(x) > {H}_{{\rm{Burg}}}(y)\,and\,{H}_{\alpha }(x) > {H}_{\alpha }(y)\,for\,all\,\alpha \in {\mathbb{R}}\backslash \mathrm{\{0\}},$$

*where Shannon, Rényi and Burg entropies are given by* *Eqs* (5), (6) *and* (7)*, respectively*.

Notice that the direct statement is a consequence of the Schur-concavity and additivity of the Shannon, Rényi and Burg entropies. Indeed, any Schur-concave and additivity function, say Φ, anti-preserves the trumping majorization relation: if $x\,{\prec }_{T}\,y$, then Φ(*x*) ≥ Φ(*y*), due to Φ(*x* ⊗ *c*) = Φ(*x*) + Φ(*c*) ≥ Φ(*y* ⊗ *c*) = Φ(*y*) + Φ(*c*).

Let us observe that the trumping majorization relation also defines a partial order. In other words, for the set of probability vectors Δ_*d*_, trumping majorization is a binary relation that satisfies (i) reflexivity, (ii) symmetry and (iii) transitivity. The reflexivity property $x\,{\prec }_{T}\,x$ is a direct consequence of the reflexivity of majorization. The symmetry property, that is, $x\,{\prec }_{T}\,y$ and $y\,{\prec }_{T}\,x$, then *x* = *y*, has been proven in refs^21,22^. Finally, the transitivity property, that is, if $x\,{\prec }_{T}\,z$ and $z\,{\prec }_{T}\,y$, then $x\,{\prec }_{T}\,y$, directly follows from the property if $x\,\prec \,y$, then $x\,\otimes \,c\,\prec \,y\,\otimes \,c$ for any *c* and transitivity of majorization. However, there is still an open question whether trumping majorization gives a lattice structure^31^. If we restrict to the case of probability vectors of dimension four and catalyst of dimension two, we are able to answer that question in a positive way by appealing to the following theorem^29^, which gives us the necessary and sufficient conditions for trumping majorization.

#### Theorem 2.

*Let x*, *y* ∈ Δ_4_*such that* $x\,\nprec \,y$. *Then*, $x\,{\prec }_{T}\,y$ *if and only if*

9 $${x}_{1}\le {y}_{1},$$

10 $${x}_{1}+{x}_{2} > {y}_{1}+{y}_{2},\,and\,{x}_{1}+{x}_{2}+{x}_{3}\le {y}_{1}+{y}_{2}+{y}_{3},$$

11 $${p}_{{\rm{\min }}}(x,y)\le {p}_{{\rm{\max }}}(x,y)$$

*with*

12 $${p}_{{\rm{\min }}}(x,y)=\,{\rm{\max }}\{\frac{{x}_{1}+{x}_{2}-{y}_{1}}{{y}_{2}+{y}_{3}},1-\frac{{x}_{4}-{y}_{4}}{{y}_{3}-{x}_{3}}\}$$

13 $${p}_{{\rm{\max }}}(x,y)=\,{\rm{\min }}\{\frac{{y}_{1}}{{x}_{1}+{x}_{2}},\frac{{y}_{1}-{x}_{1}}{{x}_{2}-{y}_{2}},1-\frac{{y}_{4}}{{x}_{3}+{x}_{4}}\}.$$

*In such a case, the catalyst has the form c* = (*p*, 1 − *p*) with *p* ∈ [*p*_min_, *p*_max_].

In the sequel, we restrict our consideration to this case.

### The lattice structure of trumping majorization for 4D vectors and 2D catalyst

Here, we consider trumping majorization for 4D probability vectors with 2D catalyst. Accordingly, in this Section, for a given *x*, *y* ∈ Δ_4_, $x\,{\prec }_{T}\,y$ means that there exists *c* = (*p*, 1 − *p*) such that $x\,\otimes \,c\,\prec \,y\,\otimes \,c$.

The next theorems establish the form of infimum and supremum.

#### Theorem 3.

*Let x*, *y* ∈ Δ_4_*. There exists x* ∧_*T*_*y such that* $x\,{\wedge }_{T}\,y\,{\prec }_{T}\,x$, $x\,{\wedge }_{T}\,y\,{\prec }_{T}\,y$ and $z\,{\prec }_{T}\,x\,{\wedge }_{T}\,y$ *for all z such that* $z\,{\prec }_{T}\,x$ *and* $z\,{\prec }_{T}\,y$. *In addition, the infimum has the form:*

14 $$x\,{\wedge }_{T}\,y=\{\begin{array}{ll}x & {\rm{if}}\,x\,{\prec }_{T}\,y\\ y & {\rm{if}}\,y\,{\prec }_{T}\,x\\ x\,\wedge \,y & {\rm{if}}\,x\,{\nprec }_{T}\,y\,{\rm{and}}\,y\,{\nprec }_{T}\,x,\end{array}$$

*where x* ∧ *y is the infimum with respect to the majorization relation*.

#### Theorem 4.

*Let x*, *y* ∈ Δ_4_*. There exists x* ∨_*T* _*y* such that $x\,{\prec }_{T}\,x\,{\vee }_{T}\,y$, $y\,{\prec }_{T}\,x\,{\vee }_{T}\,y$ *and* $x\,{\vee }_{T}\,y\,{\prec }_{T}\,z$ *for all z such that* $x\,{\prec }_{T}\,z$ *and* $y\,{\prec }_{T}\,z$. *In addition, the supremum has the form:*

15 $$x\,{\vee }_{T}\,y=\{\begin{array}{ll}y & {\rm{if}}\,x\,{\prec }_{T}\,y\\ x & {\rm{if}}\,y\,{\prec }_{T}\,x\\ x\,\vee \,y & {\rm{if}}\,x\,{\nprec }_{T}\,y\,{\rm{and}}\,y\,{\nprec }_{T}\,x,\end{array}$$

*where x* ∨ *y is the supremum with respect to the majorization relation*.

As a consequence, we have that the quadruple $\langle {{\rm{\Delta }}}_{4},{\prec }_{T},{\wedge }_{T},{\vee }_{T}\rangle$ forms the trumping majorization lattice for 4D probability vectors with 2D catalyst. In addition, we have that the trumping majorization lattice is bounded with the bottom element ${z}^{{\rm{\min }}}=(\frac{1}{4},\frac{1}{4},\frac{1}{4},\frac{1}{4})$ and the top element *z*^max^ = (1, 0, 0, 0). More precisely, it is easy to see that by considering the catalyst $c=(\frac{1}{2},\frac{1}{2})$, we have ${z}^{min}\,\otimes \,c\,\prec \,z\,\otimes \,c$ for all *z* ∈ Δ_4_. In a similar way, for the catalyst *c* = (1, 0), we have $z\,\otimes \,c\,\prec \,{z}^{\max }\,\otimes \,c$ for all *z* ∈ Δ_4_.

From the above theorems, we conclude that:

#### Corollary 1.

*Let x*, *y* ∈ Δ_4_*. Then, the following statements are equivalent:*

(i) *either* $x\,\nprec \,y$ *and* $x\,{\prec }_{T}\,y$ *or* $y\,\nprec \,x$ *and* $y\,{\prec }_{T}\,x$
(ii) *x* ∧ _*T*_*y* ≠ *x* ∧ *y*
(iii) *x* ∨_*T*_*y* ≠ *x* ∨ *y*

In other words, the catalyst is useful (in the sense that there exists a doubly stochastic matrix such that *x* ⊗ *c* = *D*(*y* ⊗ *c*) or vice versa) when infimum and supremum given by majorization differ from the corresponding infimum and supremum given by trumping majorization. This gives us an algebraic characterization of the catalyst. To illustrate these situations, let us consider the following examples.

#### Example 1.

*Let x* = (0.4, 0.4, 0.1, 0.1) *and y* = (0.5, 0.25, 0.25, 0). *It can be shown that* $x\,\nprec \,y$ *and* $x\,\nprec \,x$, *but* $x\,{\prec }_{T}\,y$ *with a catalyst c* = (*p*, 1 − *p*) *with p* ∈ [0.6, 0.625] (see ref.^29^). *Here, we have x* ∧ *y* = (0.4, 0.35, 0.15, 0.1) *and x* ∨ *y* = (0.5, 0.3, 0.2, 0), *whereas x* ∧_*T*_*y* = *x and x* ∨_*T*_*y* = *y*.

#### Example 2.

*Let x* = (0.4, 0.4, 0.2, 0) *and y* = (0.5, 0.35, 0.1, 0.05). *It can be shown that* $x\,{\nprec }_{T}\,y$ *and* $y\,{\nprec }_{T}\,x$ *(as a consequence* $x\,\nprec \,y$ *and* $x\,\nprec \,y$*)*. *Here, we have x* ∧_*T*_*y* = *x* ∧ *y* = (0.4, 0.4, 0.15, 0.05) *and x* ∨_*T*_*y* = *x* ∨ *y* = (0.5, 0.35, 0.15, 0).

### Entropic inequalities and distance on the trumping majorization lattice

Now, we show the subadditivity and supermodularity of the Shannon entropy and introduce an entropic-based distance on the trumping majorization lattice.

Let us first observe that Cicalese and Vaccaro have also shown that the Shannon entropy is subadditive and supermodular on the majorization lattice^16^. This means that,

#### Theorem 5.

*For all x*, *y* ∈ Δ_*d*_*, the Shannon entropy is subadditive, that is*,

16 $${H}_{1}(x\,\wedge \,y)\le {H}_{1}(x)+{H}_{1}(y\mathrm{).}$$

*On the other hand, the Shannon entropy satisfies the supermodular property*,

17 $${H}_{1}(x\,\wedge \,y)+{H}_{1}(x\,\vee \,y)\ge {H}_{1}(x)+{H}_{1}(y\mathrm{).}$$

Furthermore, by appealing to this last result, they have also introduced a proper distance on the majorization a lattice as follows^34^:

#### Definition 4.

*Let x*, *y* ∈ Δ_*d*_*and the distance* $D:{{\rm{\Delta }}}_{d}\times {{\rm{\Delta }}}_{d}\mapsto {\mathbb{R}}$ *such that*

18 $$D(x,y)={H}_{1}(x)+{H}_{1}(y)-2{H}_{1}(x\vee y\mathrm{).}$$

It can be shown that this definition provides a proper distance that satisfies, for *x*, *y*, *z* ∈ Δ_*d*_:

- Non-negativity: *D*(*x*, *y*) ≥ 0 with *D*(*x*, *y*) = 0 iff *x* = *y*;
- Symmetry: *D*(*x*, *y*) = *D*(*x*, *y*);
- Triangle inequality: *D*(*x*, *y*) + *D*(*y*, *z*) ≥ *D*(*x*, *z*);
- Compatibility with the majorization lattice: if $x\,\prec \,y\,\prec \,z$, then *D*(*x*, *y*) + *D*(*y*, *z*) = *D*(*x*, *z*).

Let us notice that the subadditivity and the supermodularity of the Shannon entropy can be easily extended to the trumping majorization lattice, because of the form of the infimum and supremum given in (14) and (15). That is,

#### Corollary 2.

*For all x*, *y* ∈ Δ_4_*, one has the subadditivity property of Shannon entropy*,

19 $${H}_{1}(x\,{\wedge }_{T}\,y)\le {H}_{1}(x)+{H}_{1}(y\mathrm{).}$$

*On the other hand, the supermodular property is satisfied*,

20 $${H}_{1}(x\,{\wedge }_{T}\,y)+{H}_{1}(x\,{\vee }_{T}\,y)\ge {H}_{1}(x)+{H}_{1}(y\mathrm{).}$$

So, one can intermediately define a proper distance on the trumping lattice as follows:

#### Definition 5.

*Let x*, *y* ∈ Δ_4_*and the distance* ${D}_{T}:{{\rm{\Delta }}}_{4}\times {{\rm{\Delta }}}_{4}\mapsto {\mathbb{R}}$ *such that*

21 $${D}_{T}(x,y)={H}_{1}(x)+{H}_{1}(y)-2{H}_{1}(x{\vee }_{T}y\mathrm{).}$$

This provides a proper distance in the sense that *D*_*T*_ is (a) nonnegative, (b) symmetric, (c) satisfies the triangle inequality and (d) it is compatible with the trumping majorization lattice.

## Discussion

Here, we have studied order properties of trumping majorization. In the general case, trumping majorization gives a partial order for probability vectors whose components are sorted in nonincreasing order. Moreover, by restricting to four dimensional probability vectors and two dimensional catalysts, we have shown that trumping majorization is indeed a lattice (see Theorems 3 and 4). This allows us to give an algebraic characterization of the situations in which the catalyst is useful (see Corollary 1), in the sense that one can transform a certain probability vector into another one by means of a doubly stochastic matrix.

In addition, we have proved that the subadditivity and supermodular properties of the Shannon entropy on the majorization lattice are also satisfied in the trumping majorization lattice (Corollary 2). This allows us to introduce a suitable distance among probability vectors over the trumping lattice (Definition 5). Once that one has this abstract definition, it is possible to introduce an ELOCC-based distance or a quantum catalytic incoherence-based distance specializing the input probability vectors as follows. In the first case, given the bipartite pure states *x* and *y*, with Schmidt decompositions $|x\rangle ={\sum }_{i}\sqrt{{x}_{i}}|{i}^{A}\rangle |{i}^{B}\rangle$ and $|y\rangle ={\sum }_{j}\sqrt{{y}_{j}}|{j}^{A}\rangle |{j}^{B}\rangle$ and the probability vectors of squared Schmidt coefficients *x*, *y* ∈ Δ_4_, a natural ELOCC-based distance can be defined as *D*_ELOCC_($|x\rangle$, $|y\rangle$) = *D*_*T*_(*x*, *y*). On the other side, given the pure states $|x\rangle$ and $|y\rangle$, with decompositions $|x\rangle ={\sum }_{i}\exp (\imath {\varphi }_{i})\sqrt{{x}_{i}}|i\rangle$ and $|y\rangle ={\sum }_{i}\,\exp \,(\imath {\psi }_{i})\sqrt{{y}_{i}}|i\rangle$ in a fixed reference basis {$|{i}\rangle$} and the probability vectors of absolute squared coefficients *x*, *y* ∈ Δ_4_, a natural catalytic incoherence-based distance can be defined as *D*_ICO_($|x\rangle$, $|y\rangle$) = *D*_*T*_(*x*, *y*).

Finally, we notice that, for the general case (probability vectors with dimension higher than four and catalyst with arbitrary dimension), it remains open whether trumping majorization gives a lattice structure^31^. Such characterization is important because it could lead to a better understanding not only of the ELOCC transformations^21^, but also of catalytic coherence transformations^32,33^.

## Methods

In order to prove Theorem 3, we will use the following lemma, which is valid for arbitrary dimension of the probability vectors and the catalyst.

### Lemma 1.

*Let x and y be probability vectors and let* $\underline{T}(x,y)=\{z:z\,{\prec }_{T}\,x\,and\,z\,{\prec }_{T}\,y\}$. *Then*, ∃ *x* ∧_*T*_*y if and only if* $\,\exists \,{\rm{\max }}\,\underline{T}(x,y)$, *where the maximum is taken in the sense of (standard) majorization*. *In this case, we have* $x\,{\wedge }_{T}\,y=\,{\rm{\max }}\,\underline{T}(x,y)$ *when one of the members of the equality exists*.

### *Proof*.

Let us first observe that $\underline{T}(x,y)\ne \varnothing$ because *x*∧*y* belongs to it.

Let us show that, if ∃*x* ∧_*T*_*y*, then $\exists {\rm{\max }}\underline{T}(x,y)$. On one hand, we have $z\,{\prec }_{T}\,x\,{\wedge }_{T}\,y$ for all $z\in \underline{T}(x,y)$, by definition of infimum. On the other hand, let us assume that there exists $z\in \underline{T}(x,y)$ such that $x\,{\wedge }_{T}\,y\,\prec \,z$. This implies that $x\,{\wedge }_{T}\,y\,{\prec }_{T}\,z$. Therefore, since trumping majorization is a partial order, we obtain that $z=x\,{\wedge }_{T}\,y=\,{\rm{\max }}\,\underline{T}(x,y)$.

Now, let us show the inverse statement. By hypothesis, we have $z\prec \,{\rm{\max }}\,\underline{T}(x,y)$ for all $z\in \underline{T}(x,y)$. This implies, $z\,{\prec }_{T}\,{\rm{\max }}\,\underline{T}(x,y)$ for all $z\in \underline{T}(x,y)$. As a consequence, ${\rm{\max }}\,\underline{T}(x,y)=x\,{\wedge }_{T}\,y$, this equality directly follows from the definition of infimum on the trumping lattice.□

Now, let us prove Theorem 3. We remark that this theorem is valid only for four-dimensional vectors and two-dimensional catalyst, because we will also use the Theorem 2 in order to prove it.

### *Proof*.

The cases $x\,{\prec }_{T}\,y$ and $y\,{\prec }_{T}\,x$ directly follow from the definition of infimum.

For the nontrivial case, $x\,{\nprec }_{T}\,y$ and $y\,{\nprec }_{T}\,x$, we will prove that ${\rm{\max }}\,\underline{T}(x,y)=x\wedge y$.

Now, let us look for a characterization of the vectors *x* and *y* such that $x\,{\nprec }_{T}\,y$ and $y\,{\nprec }_{T}\,x$. Let us assume (9) is satisfied, without loss of generality. Thus, we have $y\,{\nprec }_{T}\,x$. Then, we look for all the possibilities that the necessary and sufficient conditions (10) and (11) can be violated. Accordingly, we can see that, given *x*, *y* ∈ Δ_4_ such that $x\,{\nprec }_{T}\,y$ and $y\,{\nprec }_{T}\,x$, only five cases have to be considered:

(i) *x*_1_ + *x*_2_ ≤ *y*_1_ + *y*_2_, *x*_1_ + *x*_2_ + *x*_3_ > *y*_1_ + *y*_2_ + *y*_3_ and *p*_min_(*x*, *y*) ≤ *p*_max_(*x*, *y*);
(ii) *x*_1_ + *x*_2_ ≤ *y*_1_ + *y*_2_, *x*_1_ + *x*_2_ + *x*_3_ > *y*_1_ + *y*_2_ + *y*_3_ and *p*_min_(*x*, *y*) > *p*_max_(*x*, *y*);
(iii) *x*_1_ + *x*_2_ > *y*_1_ + *y*_2_, *x*_1_ + *x*_2_ + *x*_3_ > *y*_1_ + *y*_2_ + *y*_3_ and *p*_min_(*x*, *y*) ≤ *p*_max_(*x*, *y*);
(iv) *x*_1_ + *x*_2_ > *y*_1_ + *y*_2_, *x*_1_ + *x*_2_ + *x*_3_ > *y*_1_ + *y*_2_ + *y*_3_ and *p*_min_(*x*, *y*) > *p*_max_(*x*, *y*);
(v) *x*_1_ + *x*_2_ > *y*_1_ + *y*_2_, *x*_1_ + *x*_2_ + *x*_3_ ≤ *y*_1_ + *y*_2_ + *y*_3_ and *p*_min_(*x*, *y*) > *p*_max_(*x*, *y*).

By using the algorithm for the infimum, *x*∧*y*, given in^16^, it can be shown that the expression for each case has to be of the form:

22 $$x\wedge y=\{\begin{array}{cc}({x}_{1},{x}_{2},\,1-{y}_{4}-{x}_{1}-{x}_{2},{y}_{4}) & {\rm{c}}{\rm{a}}{\rm{s}}{\rm{e}}{\rm{s}}\,({\rm{i}})\,{\rm{o}}{\rm{r}}\,({\rm{i}}{\rm{i}})\\ ({x}_{1},{y}_{1}+{y}_{2}-{x}_{1},{y}_{3},{y}_{4}) & {\rm{c}}{\rm{a}}{\rm{s}}{\rm{e}}{\rm{s}}\,({\rm{i}}{\rm{i}}{\rm{i}})\,{\rm{o}}{\rm{r}}\,({\rm{i}}{\rm{v}})\\ ({x}_{1},{y}_{1}+{y}_{2}-{x}_{1},\,1-{x}_{4}-{y}_{1}-{y}_{2},{x}_{4}) & {\rm{c}}{\rm{a}}{\rm{s}}{\rm{e}}\,({\rm{v}}).\end{array}$$

Now, let us show that there does not exist a vector $z=({z}_{1},{z}_{2},{z}_{3},{z}_{4})\in \underline{T}(x,y)$ such that $x\,\wedge \,y\,\prec \,z$. This leads to our conclusion: ${\rm{\max }}\,\underline{T}(x,y)=x\,\wedge \,y=x\,{\wedge }_{T}\,y$, where the last equality directly follows from Lemma 1.

Now, we prove the nonexistence of *z* by absurd. Let us assume that there exists such vector *z*. On one hand, we obtain that *z*_1_ = *x*_1_ for all cases from the conditions $x\,\wedge \,y\,\prec \,z$ and $z\,{\prec }_{T}\,x$. This implies that *p*_max_(*z*, *x*) = 0. On the other hand, we have that *p*_min_(*z*, *x*) > 0 from $z{\prec }_{T}x$. This contradicts the necessary condition *p*_min_(*z*, *x*) ≤ *p*_max_(*z*, *x*). Therefore, such vector *z* does not exist.□

To prove Theorem 4, we will use the following lemma, which is valid for arbitrary dimension of the probability vectors and the catalyst.

### Lemma 2.

*Let x and y be probability vectors and let* $\overline{T}(x,y)=\{z:x\,{\prec }_{T}\,z\,and\,y\,{\prec }_{T}\,z\}$. *Then*, ∃ *x* ∨_*T*_*y if and only if* $\exists {\rm{\min }}\,\overline{T}(x,y)$, *where the minimum is taking in the sense of majorization. In this case, we have* $x\,{\vee }_{T}\,y=\,min\,\overline{T}(x,y)$ *when one of the members of the equality exists*.

### *Proof*.

The proof is very similar to the proof of Lemma 1. Let us first observe that $\overline{T}(x,y)\ne \varnothing$ because *x*∨*y* belongs to it.

Let us show that, if ∃*x* ∨_*T*_*y*, then $\exists {\rm{\min }}\,\overline{T}(x,y)$. On one hand, we have $x\,{\vee }_{T}\,y\,{\prec }_{T}\,z$ for all $z\in \overline{T}(x,y)$, by definition of supremum. On the other hand, let us assume that there exists $z\in \overline{T}(x,y)$ such that $z\,\prec \,x\,{\vee }_{T}\,y$. This implies that $z\,{\prec }_{T}\,x\,{\vee }_{T}\,y$. Therefore, since trumping majorization is a partial order, we obtain that $z=x\,{\vee }_{T}\,y=\,{\rm{\min }}\,\overline{T}(x,y)$.

Now, let us show the inverse statement. Let us again observe that $\overline{T}(x,y)\ne \varnothing$ because ${\rm{\min }}\,\overline{T}(x,y)$ belongs to it. By hypothesis, we have ${\rm{\min }}\,\overline{T}(x,y)\,\prec \,z$ for all $z\in \overline{T}(x,y)$. This implies, ${\rm{\min }}\,\overline{T}(x,y)\,{\prec }_{T}\,z$ for all $z\in \overline{T}(x,y)$. As a consequence, ${\rm{\min }}\,\overline{T}(x,y)=x\,{\vee }_{T}\,y$, this equality directly follows from the definition of supremum on the trumping lattice.□

Now, we will prove Theorem 4.

### *Proof*.

The proof is very similar to the proof of Theorem 3. Indeed, we have again that the cases $x\,{\prec }_{T}\,y$ and $y\,{\prec }_{T}\,x$ directly follow from the definition of supremum.

For the nontrivial case, $x\,{\nprec }_{T}\,y$ and $y\,{\nprec }_{T}\,x$, we will prove that ${\rm{\min }}\,\overline{T}(x,y)=x\,\vee \,y$. Let us again assume, *x*_1_ ≤ *y*_1_, without loss of generality. Following the cases (i)–(v) that characterize the vectors *x* and *y* such that $x\,{\nprec }_{T}\,y$ and $y\,{\nprec }_{T}\,x$ given in the proof of Theorem 3, we obtain that the expression of *x*∨*y* for each case has to be of the form:

23 $$x\vee y=\{\begin{array}{ll}({y}_{1},{y}_{2},\,1-{x}_{4}-{y}_{1}-{y}_{2},{x}_{4}) & {\rm{cases}}\,({\rm{i}})\,{\rm{or}}\,(\mathrm{ii})\,{\rm{and}}\,{y}_{2}\ge 1-{x}_{4}-{y}_{1}-{y}_{2}\\ ({y}_{1},\frac{1-{x}_{4}-{y}_{1}}{2},\frac{1-{x}_{4}-{y}_{1}}{2},{x}_{4}) & {\rm{cases}}\,({\rm{i}})\,{\rm{or}}\,(\mathrm{ii})\,{\rm{and}}\,{y}_{2}\mathrm{ < 1}-{x}_{4}-{y}_{1}-{y}_{2}\\ ({y}_{1},{x}_{1}+{x}_{2}-{y}_{1},{x}_{3},{x}_{4}) & {\rm{cases}}\,(\mathrm{iii})\,{\rm{or}}\,(\mathrm{iv})\,{\rm{and}}\,{x}_{1}+{x}_{2}-{y}_{1}\ge {x}_{3}\\ ({y}_{1},\frac{1-{x}_{4}-{y}_{1}}{2},\frac{1-{x}_{4}-{y}_{1}}{2},{x}_{4}) & {\rm{cases}}\,(\mathrm{iii})\,{\rm{or}}\,(\mathrm{iv})\,{\rm{and}}\,{x}_{1}+{x}_{2}-{y}_{1} < {x}_{3}\\ ({y}_{1},{x}_{1}+{x}_{2}-{y}_{1},\,1-{y}_{4}-{x}_{1}-{x}_{2},{y}_{4}) & {\rm{case}}\,({\rm{v}})\mathrm{.}\end{array}$$

Let us show that there does not exist a vector $z=({z}_{1},{z}_{2},{z}_{3},{z}_{4})\in \overline{T}(x,y)$ such that $z\,\prec \,x\,\vee \,y$. This leads to our conclusion: ${\rm{\min }}\,\overline{T}(x,y)=x\vee y=x{\vee }_{T}y$, where the last equality directly follows from Lemma 2.

Now, we prove the nonexistence of *z* by absurd. Let us assume that there exists such vector *z*. On one hand, we obtain that the *z*_1_ = *y*_1_ for all cases from the conditions $z\,\prec \,x\,\vee \,y$ and $y\,{\prec }_{T}\,z$. This implies that *p*_ma*x*_(*y*, *z*) = 0. On the other hand, we have that *p*_min_(*y*, *z*) > 0 from $y\,{\prec }_{T}\,z$. This contradicts the necessary condition *p*_min_(*y*, *z*) ≤ *p*_max_(*y*, *z*). Therefore, such vector *z* does not exist.□

Finally, let us prove Corollary 1.

### *Proof*.

First, note that (*i*) ⇒ (*ii*) is trivially true by the same definition of supremum. Now, we show the converse, that is, (*ii*) ⇒ (*i*). Let us assume that *x* ∧_*T*_*y* ≠ *x* ∧ *y*. By Theorem 3 we have either $x\,{\prec }_{T}\,y$ or $y\,{\prec }_{T}\,x$. In the first case, we have *x *∧_*T *_*y* = *x*. By absurdum, if we assume that $x\,\prec \,y$, then *x* ∧ *y* = *x*; thus *x* ∧_*T*_*y* = *x* ∧ *y* which is a contradiction. Hence, we have that $x\,{\prec }_{T}\,y$ and $x\,\nprec \,y$. In the second case, by using a similar argument, we obtain $y\,{\prec }_{T}\,x$ and $x\,\nprec \,x$, which concludes the proof of (*i*) ⇔ (*ii*).

By an analog argument, we can prove (*i*) ⇔ (*iii*). Finally, the equivalence between (*ii*) and (*iii*) directly follows from the equivalence relations proven above.□
